# Evaluation of an Active LF Tracking System and Data Processing Methods for Livestock Precision Farming in the Poultry Sector

**DOI:** 10.3390/s22020659

**Published:** 2022-01-15

**Authors:** Camille Marie Montalcini, Bernhard Voelkl, Yamenah Gómez, Michael Gantner, Michael J. Toscano

**Affiliations:** 1 Center for Proper Housing: Poultry and Rabbits (ZTHZ), Division of Animal Welfare, VPH Institute, University of Bern, Burgerweg 22, 3052 Zollikofen, Switzerland; yamenah.gomez@vetsuisse.unibe.ch (Y.G.); michael.toscano@vetsuisse.unibe.ch (M.J.T.); 2 Division of Animal Welfare, VPH Institute, University of Bern, Längassstrasse 120, 3012 Bern, Switzerland; bernhard.voelkl@vetsuisse.unibe.ch; 3 Gantner Pigeon Systems GmbH, 6780 Schruns, Austria; michael.gantner@gantnersolutions.com

**Keywords:** low-frequency tracking, commercial aviary, laying hens, false registrations, tree-based classifier, animal behaviour

## Abstract

Tracking technologies offer a way to monitor movement of many individuals over long time periods with minimal disturbances and could become a helpful tool for a variety of uses in animal agriculture, including health monitoring or selection of breeding traits that benefit welfare within intensive cage-free poultry farming. Herein, we present an active, low-frequency tracking system that distinguishes between five predefined zones within a commercial aviary. We aimed to evaluate both the processed and unprocessed datasets against a “ground truth” based on video observations. The two data processing methods aimed to filter false registrations, one with a simple deterministic approach and one with a tree-based classifier. We found the unprocessed data accurately determined birds’ presence/absence in each zone with an accuracy of 99% but overestimated the number of transitions taken by birds per zone, explaining only 23% of the actual variation. However, the two processed datasets were found to be suitable to monitor the number of transitions per individual, accounting for 91% and 99% of the actual variation, respectively. To further evaluate the tracking system, we estimated the error rate of registrations (by applying the classifier) in relation to three factors, which suggested a higher number of false registrations towards specific areas, periods with reduced humidity, and periods with reduced temperature. We concluded that the presented tracking system is well suited for commercial aviaries to measure individuals’ transitions and individuals’ presence/absence in predefined zones. Nonetheless, under these settings, data processing remains a necessary step in obtaining reliable data. For future work, we recommend the use of automatic calibration to improve the system’s performance and to envision finer movements.

## 1. Introduction

Tracking technologies generate sequences of chronologically ordered location data and offer a way to monitor movement of many individuals over long time periods with minimal disturbances. Tracking technologies have become valuable for detecting health issues in farm animals at an early stage [1,2,3,4] and in cage-free poultry farming, for their potential to select breeding traits that benefit welfare within cage-free systems [5,6] as well as to provide scientific information for optimal management [7]. However, cage-free housings are uniquely complex and may introduce numerous challenges for tracking technologies. For instance, cage-free housings of laying hens often contain a relatively high concentration of material that can interfere with tracking signals, including metal hardware (e.g., perches, floor, feeding lines) and multiple stacked horizontal levels that prevent direct lines of sight require by some automated tracking technologies (e.g., video tracking, infrared). Furthermore, compared to most other livestock, laying hens are relatively small animals that can be housed in large groups at very high densities, which would likely alter ultra-high frequency (UHF) radio signals [8]. Compared to most other commonly tracked livestock (e.g., swine, cattle), laying hens move differently (e.g., flying, jumping between horizontal tiers) and often faster. These challenges might induce measurement errors (as defined by the difference between a measured quantity and its true value), both of random and systematic natures [9]. Random errors are often inevitable and unpredictable, but their effects can be minimized, for example, by increasing the sample size. On the other hand, systematic errors are often predictable with consistent causes (e.g., environmental interference, improper calibration), but their effects are harder to compensate for and can lead to biases if not appropriately addressed during analysis.

Tracking systems have already been used to examine laying hens within the interior of a commercial system [10,11,12]; these tracking systems had to overcome the housing complexities described above. However, measurement errors were primarily evaluated within less complex settings (e.g., in small interior or outdoor settings) than commercial aviaries but focusing on movements of greater precision (i.e., individual location) than the current effort (transitions between predefined zones). For instance, using an ultra-wide band (UWB) system, Rodenburg et al. [6] reported an accuracy of 85% in detecting individuals’ location, and Stadig et al. [13] reported an error of less than 50 cm in 80% of measurements. These results present great potential for tracking systems to represent individual positions within free-range areas, as well as a margin to refine the data. Systematic errors were also investigated, although only within settings less complex than commercial aviaries. For instance, comparing registrations generated by a UWB system against video observations, Sluis et al. [14] observed an average overestimation of 40% of in the distance of broilers moving less than 15 m and an average underestimation of 15% in the distance of broilers moving more than 30 m. Furthermore, Stadig et al. [13] observed a larger error in certain areas of the experimental field and a negative influence of rain on the percentage of successful registrations. These results suggest that various factors, such as the individual level of activity, specific areas, and weather conditions, could cause errors in measurement. Although tracking systems within cage-free housing systems are becoming more popular, they still have challenges to overcome. We therefore studied long-term tracking in commercial aviaries at the level of visited zones (with five zones) instead of precise individual locations. In the current study, we used active tags with low-frequency (LF) tracking and UHF communication that distinguished five zones with key resources, including the three stacked tiers of a commercial aviary (top floor, nest box, lower floor), the littered floor underneath, and an outside covered winter garden. This tracking system is comparable to UWB tracking systems with lower frequencies, with the aim of reducing possible interactions with the environment, such as liquid and metallic materials [15].

To overcome measurement errors, some studies have mentioned novel placement of tracking system components [13,16], filtering of registrations that are not possible [13], or filtering of individual positions that do not move more than the 95% confidence interval of the system’s positioning errors [17]. When modifying the configuration of the tracking system is not an option, data processing may be the only alternative to refine and, in some cases, obtain validated data. Furthermore, tracking data often contain metadata associated to each registration, which could be used to detect false registrations and increase accuracy. Due to a potentially large number of available features and interaction effects, manually defining a rule-based algorithm can be time-consuming and suboptimal, whereas machine learning may offer a valuable solution for filtering false registrations. Despite potential for data refinement, there are only a few studies on UWB systems and related technologies that scrutinize data-processing methods, particularly within the unique settings of housings of cage-free laying hens. In the current study, we aimed to contribute to the collective effort of evaluating tracking systems for laying-hen farming, with a focus on the interior of a commercial aviary system. To achieve this aim, two analysis steps were involved. First, two data-processing approaches were applied to filter false registrations, including a simple deterministic approach that filters stays of short durations (SD method) and a machine learning approach (ML method) based on a tree-based classifier. The two processed datasets and the unprocessed dataset were compared against video-observation results (our gold standard). This evaluation was conducted in terms of the number of transitions per individual within the predefined zones and individuals’ presence/absence in each zone every second. Secondly, to better evaluate the tracking system, we studied the effect of filtering false registrations based on the ML method over a two-month period on 144 tracked animals under three potential influencing factors: different areas of the aviary, external temperature, and external humidity. We selected these factors because they have already shown to be associated, to some extent, with tracking-system performance and could introduce biases in our own work and that of others using comparable technology if associated with false-registrations.

## 2. Materials and Methods

### 2.1. Ethical Statement

The study was conducted according to the cantonal and federal regulations for the ethical treatment of experimentally used animals and approved by the Bern Cantonal Veterinary Office (BE-45/20).

### 2.2. Animals and Housing

**Figure 1:**
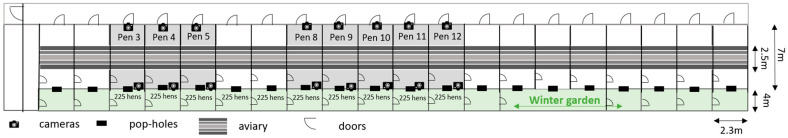
Housing setup, including the pens and aviary location in the barn, winter-garden zone, pop holes, and cameras.

As part of a larger study examining effects of on-farm hatching, approximately 4800 chicks were reared in an Inauen Natura rearing barn previously described by Stratmann et al. [18] and located at the Aviforum facility in Zollikofen, Switzerland. At seven days of age, focal animals were selected, and at approximately 16 weeks of age, all animals were transferred to an on-site commercial laying barn containing a Bolegg Vencomatic Terrace aviary. The aviary system is split into 20 identical pens separated by a vertical grid, with each pen containing 225 animals and an outside, covered winter garden that can be accessed through a pop hole (illustrated in Figure 1). Eight of the 20 pens were used for the current study, with 18 focal animals per pen (a total of 144). On the same day as the transfer to the laying barn, we mounted a tracking tag enclosed within a cloth backpack (mass: 15.6 g; height: 14.5 cm; width: 13 cm) on the back of each focal hen. These backpacks were identifiable from video cameras based on their unique colour combination.

### 2.3. Tracking System

**Figure 2:**
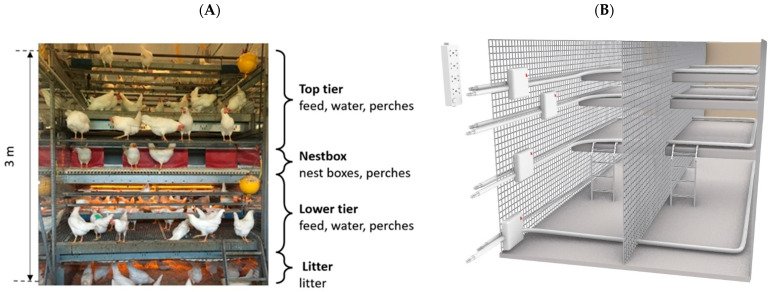
(**A**) Side view of the aviary within one pen with its three aviary zones (top tier, nest box, lower tier) and the littered floor; (**B**) simplified 3D model of the tracking system covering two pens, including the four markers of the four indoor zones and their associated cables.

To track hens across different areas within a pen, we distinguished five zones with key resources, including the three stacked tiers of a commercial aviary (top, nest box, lower), the littered floor underneath, and the winter garden, as illustrated in Figure 2A. During the laying phase, transitions between the five zones were assessed continuously for each focal hen by means of a customized tracking system. For this, three identical stations of a low-power, active tracking system (^®^Gantner Solutions GmbH, Schruns, Austria) were installed within the laying barn, each covering either two or three pens (Stations 3–5: pens 3, 4, 5; Stations 8–9: pens 8, 9; Stations 10–12: pens 10, 11, 12). Each station involved several components, including five markers (1 per zone) emitting signals through a cable (creating separately enclosed fields for each zone; Figure 2B); active tags (mass: 28.1 g) that can receive signals; and lastly, a reader that communicates through UHF (868 MHz), with the tags and a dedicated computer. 

The receiving strength of the LF signal (RSS) is used to determine theoretical distance to the antenna loop. At almost every position in any zone, the tag can receive the signal of multiple markers. This signal last for 50 ms. It is important that during that time, the tag only receives the signal of one marker; otherwise, signals would overlap and might not be valid. Therefore, the markers send at different transmission intervals (varying from 1.6 to 2.1 s depending on the zone) a fixed low carrier frequency signal of 0.125 MHz (LF-signal) that is modulated to allow markers to be differentiated. Within a 10-s interval, a tag could theoretically receive between five and six signals per marker, but this number will often be lower, as every marker has a maximum range of only two to three metres. Every time a tag receives an LF-signal, an algorithm (tag-algorithm) is applied to the registered LF signals received within the past 10 s to evaluate whether the tagged hen has transitioned to a new zone. The tag algorithm reports a new transition when a tag receives the absolute strongest signal value from the same marker twice within 10 s and if the associated zone differs from the last registered zone (pseudo-code in the Supplementary Text S1). Following the installation of the tracking-system stations, each pen was calibrated under field conditions to ensure a correct interpretation of information obtained by the devices. More specifically, a tracking tag was positioned in each of the 44 predefined critical locations per pen (e.g., where two zones border one another) to evaluate RSS against observed distance to the antenna loop and to adjust the LF signal of specific markers as necessary.

Individual transitions to a zone registered by the tracking system are hereafter called registrations. More specifically, we will refer to correct registrations (CR) for registered zones where the animal is located (i.e., true zone as determined by video) and to false registrations (FR) for registered zones not consistent with the true zone for the bird (FR). Among CRs, we distinguish two types of registrations: (1) registrations that are not associated with a true transition (corrected registrations) and (2) registrations associated with a true transition (transitional registrations). Our goal was to obtain only transitional registrations, and data processing was used towards this objective.

### 2.4. Video Observations to Detect False Registrations

Two cameras per pen were placed within the indoor portion of each pen in such a manner that each location where an animal could transition between any of the three indoor zones was visible. The view did not cover the interior of the pop hole nor the winter garden and thus did not allow transitions to the winter garden to be filmed. For the generation of the video-based tracking data as a gold standard, video data were collected over the third and fourth weeks for an 11-day period simultaneously with the collection of the tracking data. Single animals were visually tracked by two trained observers independent of one another in order to classify each registration as FR or CR. An inter-rater reliability test between the two observers for 137 registrations, including four random hens and four different days, resulted in an inter-rater reliability of perfect agreement, with all recordings classified correctly by both observers.

For the evaluation of the two processing methods (SD and ML) and the unprocessed tracking data against video-based tracking data as the gold standard, two sets of registrations were analysed through video, generating two datasets: (1) the training dataset used to develop the ML method; and (2) the test dataset used to evaluate the two processing methods, as well as the unprocessed tracking data, against video-based tracking data. As described in the Section 2.5, the training dataset was used in a cross-validation process to split the data into validation and training sets and select for the optimal models.

The training dataset was composed of 4274 registrations classified as FR or CR by means of 241 h of video observations divided into 79 batches, varying from 0.5 h to 7 h, involving 44 tracking tags over 11 days. The batches were selected based on the visual representations of individuals’ movement across all days to ensure a broad variation of movement sequences and a reasonable number of observations across zones, stations, and tracking tags. To avoid introducing noise in model training, the training dataset did not contain registrations from the winter-garden zone due to the limited camera view in the pop holes described earlier. The training dataset comprised 13% FR and 87% CR.

The test dataset was composed of 865 registrations classified as FR or CR by means of 96 h of video observation. More specifically, 48 batches (six/pen) of 2-hour video (including 47 randomly selected tracking tags) were randomly chosen over six days and reduced to 42 batches due to technical issues (e.g., backpacks not visible from the cameras). As the test dataset was used to evaluate two processing methods, including one that did not require training, the test dataset contained registrations from each of the five zones, including the winter garden. However, as the classifiers can only be tested on classes included in the training process, all registrations from the winter garden were processed solely by the SD method. Registrations in the winter garden were retained in the evaluation of both processing approaches for two main reasons: first, to avoid any bias towards poorer/greater performance of the SD method, if that zone would be more easily/laboriously detected by the tracking system compared to the other zones; second, even if the winter-garden zone is processed by the SD method when evaluating the ML method, its performance is still influenced by the ML method, typically when the ML method filters a registration to the litter zone reported between registrations in the winter-garden zone (as there would be one less transition to the winter garden). When a registration to the winter-garden zone could not be clearly classified through video observation (i.e., animal could be either in the pop hole or the winter garden), CR was used for biological relevance. We decided to define the pop-hole area (illustrated in Figure 1) as part of the winter-garden zone (and not the litter zone), as exposure to natural light in the pop hole is more similar to the winter-garden zone than the litter zone. To better evaluate the tracking system, in addition to the tracking system’s registrations, the test dataset contained all true transitions observed during video observations that were not reported by the tracking system (missed transitions). Missed transitions represented 0.6% of the test dataset. The test dataset comprised 5% FR and 95% CR.

### 2.5. Evaluation of the Two Data Processing Methods

As the tracking system used in this study evaluated the location of a tag every time the tag received an LF-signal, longer records have more opportunities for self-correction and therefore are more likely to be accurately record the location. Therefore, an intuitive and simple way to process the data is to filter all registrations that last for less than a certain threshold (SD method). We used a one-minute threshold with the objective of minimizing loss of actual transitions while maintaining a good representation of the true data.

To account for more of the available information during data processing, we used a machine learning approach (ML method) based on decision-trees, which, in addition to the registration duration used by the SD method, employed 13 features of the registrations (detailed in Table 1), including the RSS, the zone, and the station identities. The zone identities of the previous and next registrations (of the same tag) were also included to account for the movement sequence. The durations of the previous and next registrations were also included, as we expected the duration to be the most important feature for detecting FR. Our goal was to build a model to process (clean) the data rather than generate predictions about hen movement patterns. We aimed to isolate the true signal of hen movement, which can be used in future research to evaluate the drivers of hen behaviour. As such, our model is independent of external factors that could be of potential interest for future investigation (e.g., weather). Three classifiers (random forest, gradient boosting, CatBoost) based on decision-trees [19] were used to account for potential non-linearity and interaction effects [20]. The gradient-boosting classifier is a greedy algorithm that sequentially trains a shallow decision tree in order to correct the errors of the previously trained tree [21], and the CatBoost model is a recently developed gradient-boosting algorithm [22,23] that was selected in this study for its ability to process categorical features during training (algorithms of the classifiers further detailed in Supplementary Text S2). Following hyperparameter selection through a 3-fold cross-validated grid search (detailed in Table S1 of the Supplementary Materials) and model training on the training dataset, the performances of the classifiers were evaluated on the held-out test dataset using three common classifier performance measures [24]: (1) accuracy, defined as the fraction of predictions correctly classified by the model; (2) precision of class X, defined as the proportion of the predicted class X that is correctly classified by the model; and (3) recall of class X, defined as the proportion of the observed (true) class X, that is correctly classified by the model. To better contrast predictionsof the three tree-based classifiers on the test dataset in order to select one for the ML method, we used McNemar’s non-parametric test for pairwise binary classifier comparison [25] to test the null hypothesis that two models have similar proportions of errors. The normalized importance of features was generated for the selected model to understand the model’s reliance on each feature when producing its predictions. Finally, the ML method used the selected classifier to classify registrations as FR and CR and then filtered FR from the unprocessed data. However, due to the limitations of video in covering the pop-hole area, the SD method was applied here to filter registrations in the winter-garden zone.

We contrasted the two data-processing approaches by applying them to the unprocessed test dataset (i.e., including CR and FR). The resulting two processed datasets (ML and SD datasets), as well as the unprocessed test dataset, were then compared against the respective gold-standard dataset (i.e., registrations identified as CR through video observation). In each case, we evaluated two things: (1) the animal’s location (or more specifically, their presence/absence in each zone) and (2) the animal’s movement. To evaluate how well these datasets represented individuals’ presence/absence in each zone at each second, we compared their associated categorical time series (containing five categories, one for each zone). The performance was evaluated in terms of accuracy, macro-averaged recall, and macro-averaged precision (where the macro-averaged recall/precision is the average of the recall/precision across each zone). To evaluate how well these datasets represented individuals’ movement, we compared the total number of individual transitions per batch, per zone in each case. Performance was evaluated with the explained variance score (EV) and the mean absolute error (MAE), defined as:

$$EV=1-\frac{variance\left\{y_{GS}-\hat{y}\right\}}{variance\left\{y_{GS}\right\}},\;\;MAE=\frac{1}{n_{samples}}\sum_{i=0}^{n_{samples}-1}|y_{GS}{}_{i}-{\hat{y}}_{i}|$$

where $\hat{y}$ contains information from a processed dataset and $y_{GS}$ contains the respective gold-standard information. The EV is used to measure the magnitude of the expected effect on the number of transitions [26]. The MAE is used to measure, in an unambiguous and natural manner, the magnitude of the expected average error [27] in terms of the number of transitions (for a two-hour batch). This analysis was performed with Python version 3.8.5 using the SciKit Learn package [28] for the performance measures and the CatBoost package [22] for the CatBoost classifier.

### 2.6. Investigation of Influencing Factors

When comparing large datasets with thousands of hours of tracking per animal, comparison with video recordings as a gold standard becomes impractical. Therefore, to further evaluate the tracking system, we used the tree-based classifier from the ML method to identify FR (IFR) and studied the estimated error rate, defined as the number of IFRs against the total number of registrations, in relation to specific factors. The estimated error rate had a value of one when all records were filtered by the ML method and a value of zero when none was filtered. This approach has some limitations due to probable FRs not being detected or some being falsely detected. However, by removing the limitation on the number of days and individuals used, a broader investigation of the systems’ performance can be conducted. Data processing with the ML method is shown in the Results section to filter most of the true FRs (recall of class FR: 93%) and to filter mostly true FRs (precision of class FR: 84%). Therefore, IFRs should highlight most of the FRs from the unprocessed data and should be composed mainly of FRs. We applied the ML method over a two-month period, involving 144 animals, during which the hens were kept under similar management conditions every day, including 15 h of artificial light and six hours with access to the winter garden. To avoid biasing the data towards a greater error rate when the winter garden was closed, we excluded all registrations of transitions to the winter garden for periods when it was closed. We evaluated the estimated error rate in relation to different areas by reporting the mean ± SD of the estimated error rate across individuals for each of the five zones in each of the eight pens (40 pen-zone areas). We evaluated the estimated error rate in relation to external weather variables by fitting a mixed-effects logistic regression (link function: logit, R package “lme4” [29]) on the ratio of IFR to the total registrations minus IFR (per hour), with pen identity nested in station identity as a random term and hourly external humidity (%) and temperature (°C) as explanatory variables. External humidity was rescaled by dividing its values by 10. To control for variations barn management and animal behaviour throughout the day, the hour of the day was also added as a fixed effect. External humidity and temperature were obtained from the LSZB weather station (~12 km from the barn) and accessed via the Wolfram alpha API in Python. 

## 3. Results

### 3.1. Evaluation of the Two Data Processing Methods

**Figure 3:**
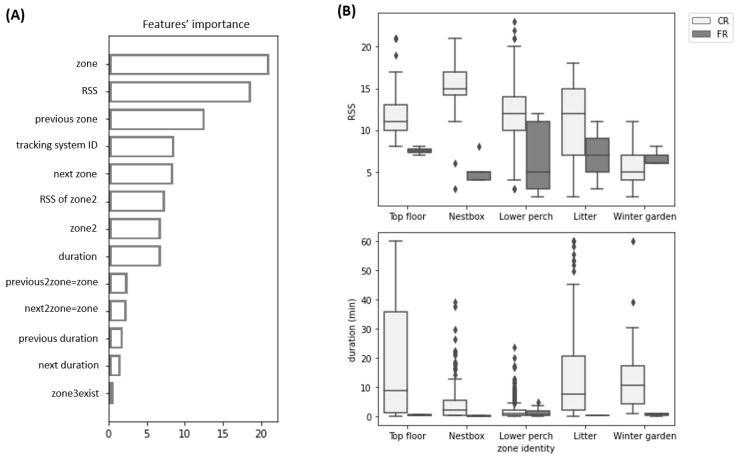
(**A**) Normalized importance of features for the selected CatBoost model. (**B**) Box plots of the RSS (top) and duration of stay (bottom) of the test dataset registrations, split into CR (light grey) and FR (grey), as produced by video observations, are displayed for each zone.

On the test dataset, the three classifiers showed stable (over 100 random seeds) accuracy, recall, and precision (Figure S1 of the Supplementary Materials), and the McNemar’s test showed a similar proportion of errors between each classifier (*p* > 0.05). Thus, with an accuracy of 99%, we selected the CatBoost algorithm for the ML method because of its ability to handle categorical variables in Python. Additionally, 84% of the time that the model identified an FR, the model prediction was correct (precision of class FR). and 100% of the time that the model identified a CR, the model prediction was correct (precision of class CR). Additionally, 93% of the FR observations were classified by the model as FR (recall of class FR), and 99% of the CR observations were classified by the model as CR (recall of class CR). The zone identity, RSS, and the previous registrations’ zone identity were the three most important features, accounting for 21%, 19%, and 13% of the overall importance of the features, respectively, while duration accounted for 7% (Figure 3A). To further illustrate the importance of the features, Figure 3B show the RSS and duration of the test dataset’s registrations, split into CR and FR (from video observations). The receiving strength of the LF signal was generally higher for the correct registrations of all indoor zones. We also observed longer duration of stay to be more frequent among the correct registrations, with the exception of registrations in the lower perch zone, where no difference in the duration of stay was observed between correct and false registrations.

**Figure 4:**
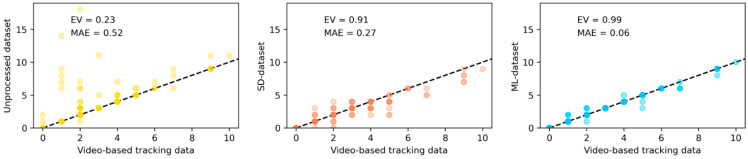
Number of transitions per individual (per batch, per zone) for the unprocessed, SD and ML datasets against video-based tracking data and associated *EV* and *MAE* scores. Overlapping data points are represented by darker shading.

The unprocessed, SD and ML datasets all determined an individual’s zone (every second), with an accuracy of 99%, 98%, and 100%, respectively, and displayed the same values (99%, 98%, and 100%, respectively) for the macro-averaged precision and macro-averaged recall. We found the ML and the SD datasets to underestimate the number of transitions by an average 0.27 and 0.06 transitions per zone, respectively, for a two-hour batch, in contrast to the unprocessed dataset, which overestimated the number of transitions by approximately 0.5 transitions per zone, on average, for a two-hour batch (average number of transitions per batch, per zone by video observation was 1.8). The percentage of variance of the ground-truth data recovered by the unprocessed, SD and ML datasets was 23%, 91%, and 99%, respectively, which is further illustrated in Figure 4.

### 3.2. Investigation of Influencing Factors

**Figure 5:**
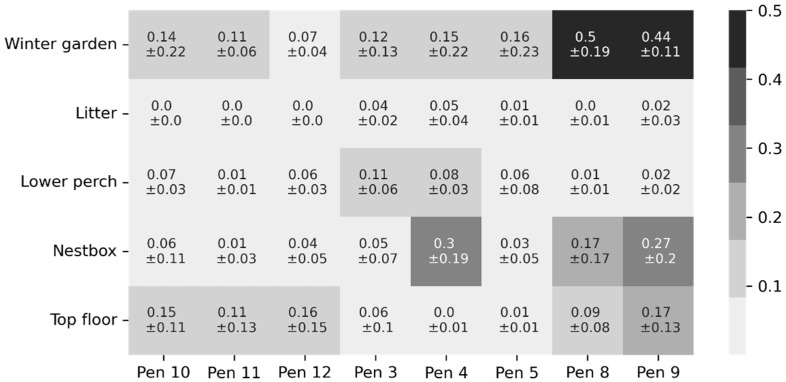
Estimated error rate (mean ± SD) for pen-zone areas.

**Figure 6:**
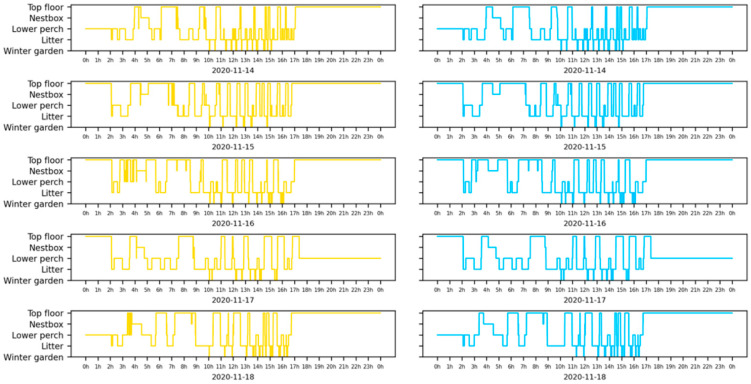
Unprocessed data (yellow) and processed data by the ML method (blue) of a single animal over five consecutive days. Each row represents a single 24-hour day, with the zone identities represented on the y-axis (the indoor zones are ordered following the aviary order, and the winter garden is represented below the indoor zones).

The estimated error rate across pen-zone areas varies from 0.0 ± 0.0 (e.g., litter area within each pen of Stations 10–12) to 0.5 ± 0.19 for Pen 8, suggesting that half of the registrations in the winter garden from Pen 8 were filtered by the ML method. The estimated error rate per pen-zone area is further detailed in Figure 5. Furthermore, we found a negative effect of humidity (*p* = 0.003) on the estimated error rate, with an odds ratio of 0.96 (95%-CI [0.94, 0.99]), indicating a 4% lower likelihood of obtaining a false registration with an increase in humidity of 10%. Additionally, we found a negative effect of temperature (*p* < 0.001) on the estimated error rate, with an odds ratio of 0.97 (95%-CI [0.96–0.98]), indicating a 3% lower likelihood of obtaining a false registration with an increase in temperature of 1 °C (for further details, see Table S2 of the Supplementary Materials). The difference between the unprocessed and the processed data (by the ML-method) is further illustrated in Figure 6 through a visual representation of an animal’s transitions over eight consecutive days. For instance, observed several transitions filtered by the ML method between the lower-perch and top-floor zones.

## 4. Discussion

We found the presented LF tracking system accurately determined the presence of animals in a given zone (at the second level), with macro-averaged precision, and macro-averaged recall of 99% when compared against video observations of the test dataset. This good performance might be explained by the tag algorithm, which searches for new transitions, on average, every 0.5 s (i.e., each time a tag receives an LF signal), thus regularly providing opportunities for correctional records. However, the number of transitions in a zone generated by the tracking system was overestimated and only explained 23% of the true variance (as observed by video). Therefore, the unprocessed tracking data did not constitute a good representation of individual transitions between the five zones, which could be emphasized by the observed differences in the estimated error rate within specific pen-zone areas. On the one hand, we observed clear differences in the estimated error rate of a given zone across different stations (e.g., winter-garden zones in Stations 10–12, Stations 3–5, and Stations 8–9 had a mean estimated error rate varying, across their respective pens, between 0.07 and 0.14, 0.12 and 0.16, and 0.44 and 0.5, respectively). On the other hand, we observed differences within pens of the same station (e.g., nest-box zone in Stations 3–4 had an estimated error rate of 0.05 ± 0.07 in Pen 3, 0.3 ± 0.19 in Pen 4, and 0.03 ± 0.05 in Pen 5). The observed differences in the estimated error rate across different pen-zone areas aligned well with locations described through anecdotal notes made during video observations describing precise locations where a tracking tag generated a high amount of FR (by repeatedly switching between two, sometimes non-neighbouring zones) while the animal was immobile (weak spots). An explanation for the existence of weak spots may be the pen furnishing blocking the line of sight between tags and signal cables, which is known to cause signal interference in UWB systems [16]. More specifically, metallic materials can absorb the signal and distort the electromagnetic field, which could either block or enhance the signal, rendering RSS a poor representation of the distance to the signal cable, possibly explain errors between non-neighbouring zones. Our tracking system was designed to use a lower frequency than a common UWB system in order to avoid possible interactions with metallic materials, although signals may still be affected. 

Furthermore, the existence of weak spots may be attributed to the calibration process, a manual and time-consuming step performed independently for each station and iteratively through each pen. When a tracking tag was detected in an incorrect zone during the calibration process, the LF values of specific markers were adjusted. As the pens are steel cages, the LF field generated by each marker can be slightly inhomogeneous. As a result, when an LF signal value is adjusted, all measurements must be repeated to ensure that the change in the LF value did not lead to further detection errors. The difficulty lies in setting the LF values of the markers in such a way that the correct zone is detected in all locations of the tracking system. In particular, the nest-box zone is a small zone located between two zones (Figure 2), and a change in the LF value of the marker had a greater effect on the neighbouring zones because it quickly led to the tracking tag being detected in the incorrect zone. Therefore, we recommend the use of an automatic calibration process to improve the system’s performance. To achieve this, within each zone, several tags would be placed at predefined locations of critical measurement points. Each signal strength received by any tag from any marker would be registered. An algorithm would be executed every 10 s (ensuring enough time for adjustment of LF signal values to take place in the field) on all RSS registered within the past 10 s. This algorithm would identify the most problematic zone, defined, for example, by the zone with the smallest dB difference in relation to another zone (across all tags in that zone). If this difference does not exceed the limit of 1 dB in relation to another zone, the LF signal value of the associated marker is automatically adjusted. As soon as 100 consecutive runs induce no adjustment of an LF signal, the calibration is complete. An automatic calibration would save time as only one person would work on the calibration. This would also offer new opportunities, such as smaller zones, allowing for registration of finer movements. For instance, in our settings, it might be possible to differentiate between the nest boxes and the balcony in front the nest boxes (currently, both are registered as the nest-box zone). Furthermore, automatic calibration would ensure more homogeneous LF values across markers from the same zone across all pens, and consequently, more comparable datasets across different stations and pens would be generated.

Our tracking system’s poor performance in representing individual transitions highlights the importance of processing automatically generated datasets. Relevant data-processing studies are lacking, although they could help to standardize this process to generate comparable datasets across different studies. The benefit of this work is most essential in light of rapid development in technology in order to manage and improve the welfare of animals within commercial livestock systems [1,2,30,31,32,33,34,35]. We showed that the data processed by a simple filtering of registrations associated with short durations (<1 min) of stays was suitable for monitoring the number of transitions per individual per zone, accounting for 91% of the actual variance (as observed by video). We further reported a gain in performance using a tree-based classifier to filter false registrations, accounting for 99% of the true variance in the number of transitions per individuals, which could partly be explained by the additional information provided to the ML method. Indeed, zone identity and RSS were the two main features upon which the tree-based classifier based its predictions, while the SD method was based solely on the duration of the stay. Interestingly, this also suggests that our expectation of the record’s duration being the most important feature to detect FR was incorrect when other features are included. The current study did not allow for this comparison when a single feature is used; however, further studies using a simple rule-based approach should consider the RSS addition to the records’ duration. The importance of features further suggests that the zone identities of the previous and next registered record are of greater importance than the duration of stay from the previous and next registrations. Results concerning the importance of can offer direction on how to improve similar tracking systems, for instance, by including a threshold of RSS values for each zone based, for example, on the result of an automatic calibration. Another possibility would be to include the SD method as part of the tag algorithm, although this would eliminate the possibility of registering fast transitions between two zones (<1 min).

The ML method required additional efforts, for instance, more video observations, compared to the SD method and statistical modelling in R or Python. Therefore, the choice between both methods relies on a compromise between time accorded in data processing and the performance of the processed data. In the current study, the SD method recovered 8% less of the true variance in the number of transitions per individuals than to the ML-method. To put this value in context, we used simulated sampling to estimate the impact of a comparable loss on the effect size (measured by the Pearson correlation) of a simulated movement variable, M, on a simulated health variable, H. The two simulated variables (M and H) followed a standard normal distribution, with a Pearson correlation coefficient varying from 0.15 to 0.40 to cover potentially interesting ranges of effect sizes when studying movements in relation to health [36,37] and heritability of behaviour [38,39,40]. By adding noise to M (and calling the result M’), we estimated (over 10,000 simulations, per sample size) the percentage of cases where significance would be lost (*p* > 0.05), depending on the initial effect size and sample size. Our estimations suggest that a change in percentage of the initial variance explained by M’ from 0.99 to 0.91 would change the significance of a critical test in 26% (or 25%) of cases when applying a sample size of 80 (or 120) and an initial effect size of 0.25 (or 0.2), respectively (see details in Figure S2 of the Supplementary Materials). Therefore, using a tree-based classifier to filter false registrations can be greater value for studies with small sample and effect sizes (e.g., *n* = 120, effect size of 0.2) than the filtering approach using stays of short duration as threshold. For large sample sizes or samples with strong correlations between the measured movement and the trait of interest, the SD method might produce equally reliable results as the ML method.

Our results further reported a marginal effect of periods of time characterized by higher humidity or higher temperature, associated with a lower estimated error rate of transitions to the winter garden. Because air is our medium of signal transmission, when humidity is changes, the magnetic field is also expected to change. As calibration was conducted in August 2020, the performance of the tracking system may be optimized for a period with higher temperature than average. Additionally, as Richards et al. [41] reported, associations between daily weather conditions and mean pop-hole usage in laying hens, including an increase in mean pop-hole usage associated with an increase in temperature, and the influence of the weather conditions on animal behaviour may be explanatory. In spite of these results, external environmental factors cannot be controlled for and are part of the experiment. However, these results can aid in interpretation and awareness of possible limitations for subsequent analyses of these or similar tracking data.

## 5. Conclusions

The active LF tracking system evaluated in this study determined the presence/absence of birds in a zone with an accuracy of 99% but overestimated the number of transitions by birds per zone, explaining only 23% of the true variation (as observed by videos). However, we showed that filtering stays of short durations rendered the data suitable for monitoring the number of transitions per individual, explaining 91% of the true variation, and that the use of a tree-based classifier to filter false registrations recovered an additional 8% of the true variation. Simulations further suggested that a machine learning approach for data processing could be of greater value than a simple deterministic approach in studies with small sample and small effect sizes. Results also suggest that filtering false registrations may reduce the effect of systematic errors towards certain pen-zone areas and towards periods of time characterized by lower humidity or temperature values. However, results also suggest that these factors might, to some extent, remain in the processed data and should be considered properly in subsequent analyses. In conclusion, this tracking system is well suited for complex indoor housing (similar to commercial aviaries) to measure the transitions of individuals and the presence/absence of birds in predefined zones (thus, duration of stays in zones). Nonetheless, under these settings, data processing remains a necessary step in obtaining reliable tracking data. For future work, we recommend the use of automatic calibration to improve the system’s performance and to envision finer movements.

## Figures and Tables

**Table 1:** Record features used to train the model and the normalized importance of features in the final CatBoost model.

Feature Name	Description
previous zone; zone; next zone	zone identity of the previous/considered/next registered record with the strongest LF signal, indicating the zone where the individual has transitioned/is transitioning/will transition to
RSS	a measurement of the power present in the strongest received LF signal (dB)
tracking system ID	identity of the tracking-system copy
previous duration; duration; next duration	reported time of stay in the zone from the previous/considered/next registered record
zone2	second zone identity with the strongest LF signal
RSS of zone2	a measurement of the power present in the second strongest received LF signal (dB)
zone3exist	binary feature that equals 1 if the tag registers a signal of at least three different zones during the last 10 s, and otherwise equals 0
next2zone = zone; previous2zone = zone	binary feature that equals 1 if the registered second zone from the next/previous record is the same as the occurring zone, and otherwise equals 0

## Data Availability

The full code and video observations results (test and training datasets) can be found on a public GitHub repository (https://github.com/cam4ani/PhD-AnimalWelfare/tree/main/Chapter0-GantnerCleaning (accessed on 12 October 2021)).

## Supplementary Materials

The following are available online at https://www.mdpi.com/article/10.3390/s22020659/s1, Table S1: Selected grid-search parameters for the random forest, gradient boosting, and CatBoost classifiers. When a parameter is not applicable for a specific classifier, the “-” notation is used. Table S2: Output of the logistic regression with the proportion of IFR to the number of registrations minus IFR as response variable and the humidity, temperature, and hour of day as fixed effects. Figure S1: Precision per class (0: FR; 1: CR), recall per class (0: FR; 1: CR), and accuracy of the three classifiers over 100 random seeds. Figure S2: Percentage of simulations that lost significance (*p* > 0.05) of associated initial effect size (measure by Pearson correlation between two simulated samples from a normal distribution: M’ and H) after a change in percentage of the true variance recovered by M’ from 0.99 to 0.91, depending on the initial effect size (varying from 0.16 to 0.4) and sample size (varying from 80 to 280).
