# Supplementary material for: Evaluation of an Active LF Tracking System and Data Processing Methods for Livestock Precision Farming in the Poultry Sector
Source: Sensors (Basel). 2022 Jan 15;22(2):659. doi: 10.3390/s22020659 (PMC8780220; doi:10.3390/s22020659)
Supplement: Supplementary file 1 [file sensors-22-00659-s001.zip › sensors-1441836-supplementary.pdf]

Supplementary materials

Supplementary Tables

Table S1. Selected grid-search parameters for the random forest, gradient boosting and catboost classifiers. When a parameter does not apply for a specific classifier the "-" notation was used.

Parameter name (from python package)	brief description	random forest	gradient boosting	Catboost
max_depth	maximum depth of the tree	11	5	6
n_estimators	number of trees in the forest	80	60	500
min_samples_split	minimum number of samples required to split an internal node	3	8	
min_samples_leaf / min_data_in_leaf (Catboost)	minimum number of samples required to be at a leaf node	2	2	1
min_impurity_decrease	threshold at which a node will be split if this split induces a decrease of the impurity greater than or equal to this value	0.001	0.0005	-
criterion	function used to measure the quality of a split	entropy		-
learning_rate	rate at which the contribution of each tree is shrink by	-	0.15	-
max_features	number of features to consider when looking for the best split	-	sqrt	-
n_iter_no_change	number of iterations used to decide if early stopping will be used to stop training when validation score is not improving by at least 0.001	-	8	-
od_wait	number of iterations to continue the training with the optimal metric value	-	-	10
l2_leaf_reg	coefficient at the L2 regularization term of the cost function	-	-	2

Table S2. Output of the logistic regression with the proportion of IFR to the number of registrations minus IFR as response variable and the humidity and temperature as fixed effects.

	cbind(IFR, nbr.registrations - IFR)		
Predictors	*Odds Ratios*	*CI*	*p*
(Intercept)	0.47	0.14 – 1.61	0.229
humidity_divided_by_10	0.96	0.94 – 0.99	**0.003**
temperature_C	0.97	0.96 – 0.98	**<0.001**
hour	0.97	0.95 – 0.99	**0.001**
**Random Effects**			
σ^2	3.29		
τ_{00} PenID:system	0.07		
τ_{00} system	1.05		
Marginal R^2 / Conditional R^2	0.006 / 0.043		

Supplementary Figures

Figure S1. Precision per class (0: FR; 1: CR), recall per class (0: FR; 1: CR) and accuracy of the three classifiers over 100 random seeds.

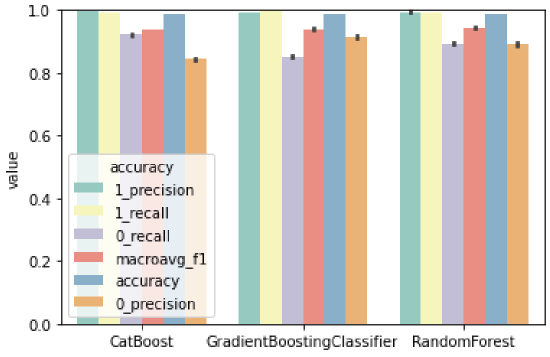

Figure S2. Percentage of simulations that lost significance ($p>0.05$) of its associated initial effect size (measure by pearson correlation between two simulated samples from a normal distribution: M′ and H) after a change in percentage of the true variance that is recovered by M′ from 0.99 to 0.91, depending on the initial effect size (varying from 0.16 to 0.4) and sample size (varying from 80 to 280).

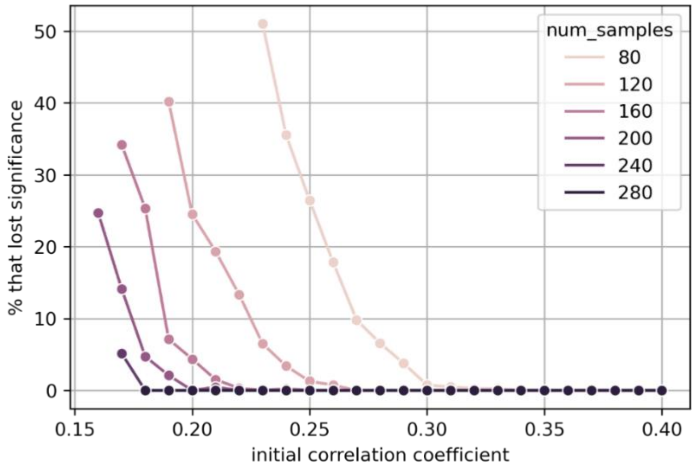

Supplementary Text

S1. Pseudo-code of the tag-algorithm

START every time a tag receive an LF-signal (=markers signal) (it can be up to every half seconds in average i.e. 2sec/4markers)

List = all received signals sent from any markers of past 10 sec of the associated tracking system
previous-zone-logged = last logged transition
previous-dB-zone = dBs of last logged zone

current-dB-of-highest-zone = maximum dB entry in List
current-highest-zone = zone with maximum dB entry in List (find thanks to the modulated signal)

If current-dB-of-highest-zone is not the unique maximum of the List:
Don't register a new transition and repeat the procedure

current-dB-of-second-highest-zone = second highest dB entry in List
current-second-highest-zone = zone with second highest dB entry in List

If current-highest-zone ≠ current-second-highest-zone:
Don't register a new transition and repeat the procedure

If previous-zone-logged ≠ current-highest-zone:
Register a new transition to current-highest-zone and repeat the procedure

END

S2. Tree-based classifiers: In this study we used three decision-tree based classifiers. A decision tree is a non-parametric supervised learning method that performs recursive partition of the instance space (Rokach & Maimon, 2005). Typically, a decision tree is an acyclic directed graph and has one node with no incoming edges (root node) as well as nodes with one incoming edge. All nodes with at least one outcoming edge are called internal nodes and all nodes with no outcoming edge are called decision nodes. During training, each internal node aims to split the instance space while optimizing the classifier's performance. The Gradient boosting classifier is a 'greedy' algorithm that sequentially trains a shallow decision tree in order to correct the errors of the previously trained tree (Friedman, 2001). The

Catboost method is a recently developed gradient boosting algorithm (Dorogush et al., 2017; Prokhorenkova et al., 2017) that we selected in this study for its ability to process categorical features during training. More specifically, it substitutes each categorical feature with a numerical feature by using an ordered target encoding method. Target encoding commonly replace the category x_k^i of the kth training example of the ith categorical feature, with the estimated expected target value *y* conditioned by this category: $\mathbb{E}(y|x^i = x_k^i)$. Target encoding is known to suffer from target leakage and CatBoost tries to overcome this issue. For that purpose, it adds an artificial timeline to the training dataset by permutating the set of observation in a random order and computes each encoding value based on its own artificial history only (Prokhorenkova et al., 2017). As samples with a shorter history will have a target encoding value with higher variance, CatBoost used several random permutations during training.

References

Dorogush, A. V., Ershov, V., & Gulin, A. (2017). CatBoost: gradient boosting with categorical features support. *Advances in Neural Information Processing Systems*, 1–7. http://arxiv.org/abs/1810.11363

Friedman, J. H. (2001). Greedy function approximation: a gradient boosting machine. *The Annals of Statistics*, *29*(5), 1189–1232. https://doi.org/10.1214/aos/1013203451

Prokhorenkova, L., Gusev, G., Vorobev, A., Dorogush, A. V., & Gulin, A. (2017). CatBoost: unbiased boosting with categorical features. *Advances in Neural Information Processing Systems*, *2018-December*, 6638–6648. http://arxiv.org/abs/1706.09516

Rokach, L., & Maimon, O. (2005). Decision Trees. In O. Maimon & L. Rokach (Eds.), *Data mining and knowledge discovery handbook* (pp. 165–192). Springer. https://doi.org/10.1007/0-387-25465-X_9
